# Digital transformation of health quality services in the healthcare industry during disruption and society 5.0 era

**DOI:** 10.3389/fpubh.2022.971486

**Published:** 2022-08-04

**Authors:** Kameswara Natakusumah, Erna Maulina, Anang Muftiadi, Margo Purnomo

**Affiliations:** Department of Business Administration, Faculty of Social and Political Sciences, Universitas Padjadjaran, Bandung, Indonesia

**Keywords:** digital transformation, health quality services, healthcare industry, disruption, society 5.0 era

## Introduction

Digital transformation has played a role in revolutionizing various industries, especially in the health sector. Technology in the health sector allows an individual to have a healthier life, a longer life expectancy, and a more productive life. For example, in 2015, telemedicine was accessed by more than one million people. This figure will increase significantly in 2021, where the number of people who access telemedicine has reached 12 million people. According to Tortorella et al. (1) technology has empowered patients even in remote areas to access quality health services.

According to Maiurova et al. (2), Pappas et al. (3), Ricciardi et al. (4), and Tortorella et al. (1), in addition to telemedicine, several other health technologies in the industrial era 4.0 that have been developed and utilized by various service facilities include artificial intelligence, blockchain, IoT (internet of things), and robotic services. Many health companies view technology not only as an infrastructure but also as a strategic asset. From this fact, the idea arises that optimally utilized technology will provide insight or input that is very useful for business progress. Appropriate data analysis can be used to improve service access to the community, increase the effectiveness of human resources, improve service quality, and reduce health care costs.

According to Maiurova et al. (2), Pappas et al. (3), Ricciardi et al. (4), and Tortorella et al. (1), the use of health technology among consumers also opens up opportunities for patients and their families, so that it is easier to get information and understanding about diseases, treatment options, and to easily access and choose hospitals or health facilities that suit their needs. By realizing the benefits of this digital transformation, more and more companies operating in the health sector, including hospitals, are taking the initiative to adopt this digital transformation into their management systems to produce better quality health services. According to Kruszyńska et al. (5) and Clinker et al. (6) not all health facilities are ready to welcome the era of disruption 4.0 which is full of digitization. According Gopal et al. (7) in today's digital era, patients have started to focus on various obstacles related to human resources, funding sources, business processes, government regulations and regulations, as well as the absence of a data integration system are often challenges in realizing this. These are relevant ministries, professional associations, and implementing doctors to be able to always collaborate and be open to the process of renewal and learning. The Ministry of Health always supports efforts to digitize hospitals, which is shown in various existing innovations, including smart e-health concepts such as telemedicine, and e-medical records. Kraus et al. (8) the digitalization requires clear regulations and supports the growth of the system with one goal, namely improving the quality of Indonesian public health services.

## Discussion and opinion

The healthcare industry is entering an era of digital innovation where patients are looking for services that can directly answer their needs because they are limited by their daily activities. Consumers who are looking for medical information on the internet, looking for information about doctors, booking a schedule of health checks. Based on these facts, it is necessary for the hospital management team to find out the needs of target consumers or patients and incorporate them into a digital system (e.g., ease of access using a smartphone). This market need is being exploited by several health technology companies, which are currently growing in the community. According to Klinker et al. (9), Marques and Ferreira (10), Maiurova et al. (2), Pappas et al. (3), Ricciardi et al. (4), and Tortorella et al. (1), big data combines very large amounts of information and various formats, namely from the use of social media, e-commerce, online transactions, financial transactions, as well as identifying trends and business patterns in the future. According to Maiurova et al. (2), Pappas et al. (3), Ricciardi et al. (4), and Tortorella et al. (1), in the healthcare industry, big data can provide several advantages, including lower medical error rates, facilitating preventive healthcare, and more accurate predictions for recruiting human resources (e.g., by helping hospitals and clinics predict an increase in the number of patients over a given period of time). Hermes et al. (11) thereby helping management decide to increase the number of staff at that time. In addition to the need for investment in the field of big data, processing and analysis of the data is also needed to identify business weaknesses and help management to better understand the intended target patient.

In today's digital era, patients have started to focus on preventive health and are more concerned about knowing various things related to medical information. The implication is that several companies have invested in the field of medical devices that can be used by patients to determine their health status. Existing medical devices include heart rate detectors, exercise trackers, sweat discharge measuring devices, tools to measure blood sugar levels, and oxygen levels. According to Filgueiras et al. (12), Maiurova et al. (2), Pappas et al. (3), Ricciardi et al. (4), and Tortorella et al. (1), big information gathered from big data and other sources (such as social media) can help companies to develop health recommendation services to patients. This is what is called predictive health care, where we can now predict what diseases and disorders may become epidemic in the future. Health facilities can certainly anticipate the estimated disease or outbreak that will occur, and prepare the necessary prevention or handling steps.

In addition, according to Buton-Jones et al. (13), the negative impact of digital culture in healthcare can be observed from digital security, especially regarding personal data and privacy. Because digital culture has opened up opportunities for crimeby using other people's personal data for profit. Faddis (14) digital fraud often occurs because of the misuse of personal data by individuals with advanced digital technology. Digital transformation in healthcare during the COVID-19 pandemic, it was getting stronger along with the importance of the role of information technology in strengthening all daily activities of people in Indonesia. Digital transformation is a change related to the application of digital technology in all aspects of people's lives. Digital transformation in healthcare includes the use and capabilities of informing digital awareness to the public. The stages in digital transformation in healthcare are the stages of using digital processes in healthcare that allow innovation and creativity in one particular digital product.

## Conclusion

In the digital era and disruption 4.0, there are still many hospitals and health care facilities that face the challenges of a lack of openness, motivation, and good knowledge management from hospital management, medical service doctors, and information technology teams in organizations that also need to be addressed. Hospitals need to be motivated to immediately apply information technology in their management in order to realize optimal data integration on a national scale. The problem of using big data, data security and protection, data privacy, and the use of cloud computing systems is also one of the issues that is quite challenging to understand and apply in business. Various recommendations to related parties have been formulated in the focus group discussion. Recommendations are addressed to the government, especially those authorized to issue regulations and financial support, as well as hospital management to increase implementation commitment, knowledge management of big data analysis and cloud systems, as well as empowering human resources within organizations. These recommendations are expected to be the first step in realizing a digital-based health system that is able to provide quality health services for the community.

## Author contributions

All authors listed have made a substantial, direct, and intellectual contribution to the work and approved it for publication.

## Conflict of interest

The authors declare that the research was conducted in the absence of any commercial or financial relationships that could be construed as a potential conflict of interest.

## Publisher's note

All claims expressed in this article are solely those of the authors and do not necessarily represent those of their affiliated organizations, or those of the publisher, the editors and the reviewers. Any product that may be evaluated in this article, or claim that may be made by its manufacturer, is not guaranteed or endorsed by the publisher.
